# Identification and phylogenetic analysis of the mitochondrial genome of *Hemibarbus labeo* BML (Cypriniformes: Cyprinidae)

**DOI:** 10.1080/23802359.2020.1778570

**Published:** 2020-07-06

**Authors:** Yuting Sun, Qilin Wang, Jiangfeng Ren, Shoubao Yang

**Affiliations:** a College of Life Sciences, Shaoxing University, Shaoxing, P. R. China; b Chunhui Senior High School, Shangyu, P. R. China

**Keywords:** Mitochondrial genome, *Hemibarbus labeo* BML, identification, phylogenetic analysis

## Abstract

The complete mitochondrial genome of *Hemibarbus labeo* BML (Cypriniformes: Cyprinidae) is 16,612 bases in length. It consists of 22 tRNA genes, 13 protein-coding genes, 2 rRNA genes, and 1 non-coding region. Its overall nucleotide composition is A: 29.85%, G: 17.07%, T: 25.86%, and C: 27.23%, respectively, with an A + T rich feature (55.71%). The gene arrangement and organization of the mitogenome from *H. labeo* BML were very similar to other Cyprinidae fishes. The phylogenetic analysis showed that *H. labeo* BML clustered in genus Hemibarbus. These results will contribute to the taxonomy and conservation biology studies of *H. labeo.*

The *Hemibarbus labeo* (Cypriniformes:Cyprinidae) is a small benthic fish distributed widely in China (Xu et al. 2007; Lian et al. 2012; Wu 2015; Lan et al. 2016; Gao et al. 2017). It is artificial cultured (Xu et al. 2009; Li et al. 2015; Zhang et al. 2015; Luo et al. 2016), and used as a commercially important fish because of its tender meat and high nutrition (Gu et al. 2006; Lu et al. 2007; Li et al. 2011; Lian et al. 2011; Lv et al. 2015; Chen et al. 2019; Wei et al. 2020).

The Baima Hu Lake, a small famous lake in Shangyu district, Shaoxing city, East China. The wild population of *H. labeo* in this Lake declines rapidly because of increasing capture pressure and dredging works.

Sequencing and identify the complete mitogenome and analyze its phylogenetic relationships with the related species are necessary for the taxonomy and sustainable utilization of *H. labeo*. In the present study, the complete mitochondrial genome was sequenced and identified from an individual of *H. labeo* sampled from the Baima Hu Lake of eastern China (33°13′47.7″N, 119°08′49.4″E), and was kept in 99% ethanol in the Aquatic Service Platform of Shaoxing (accession no. SXAF20200512). The genomic DNA was extracted and used as template.

Comparison of mitogenome data could be a powerful tool for taxonomy and conservation biology studies (Min and Park 2009; Chen et al. 2013; He et al. 2014).

The complete mitogenome sequence of *H. labeo* BML (GenBank accession no. MT478137) was determined to be 16612 bp in length. It consists of 22 tRNA genes, 13 protein-coding genes (PCDs), 2 rRNA genes, and 1 control region. The gene arrangement and organization of all encoded genes of the mitogenome from *H. labeo* BML were very identical to other Cyprinidae fishes (He et al. 2014; Wang et al. 2015).

The overall nucleotide composition of the *H. labeo* mitochondrial genome is A: 29.85%, G: 17.07%, T: 25.86%, and C: 27.23%, respectively, which shows an A + T rich feature (55.71%) as that of other vertebrate mitochondrial genomes (Tzeng et al. 1992; Jondeung et al. 2007; He et al. 2014). All of the genes were encoded on the heavy strand (H-strand), except one PCG (*ND6*) and eight tRNA genes (tRNA^Ala^, tRNA^Asn^, tRNA^Cys^, tRNA^Gln^, tRNA^Glu^, tRNA^Pro^, tRNA^Ser(UCN)^, and tRNA^Tyr^).

The complete mitogenome sites have 106 variable sites between *H. labeo* BML and *H. labeo* (GenBank accession no. KP064328), accounted for 0.64% of the complete mitogenome sites, these base variation were detected not only in various PCD genes but also in the non-coding region (D-loop).

Based on the complete mitochondrial genome sequences of *H. labeo* BML and other Cyprinidae fishes, a phylogenetic tree was constructed by the NJ method (Figure 1). The mitogenome of *H. labeo* BML shows more closer relationship with other Hemibarbus fishes including *H. labeo* (Kim et al. 2009), *H. barbus*, *H. maculatus*, *H. medius*, *H. umbrifer*, *H. mylodon*, and *H. longirostris.* These results showed that, the mitogenome sequence can provide useful information in the taxonomy and conservation biology studies of *H. labeo* and other fish species.

**Figure 1.**
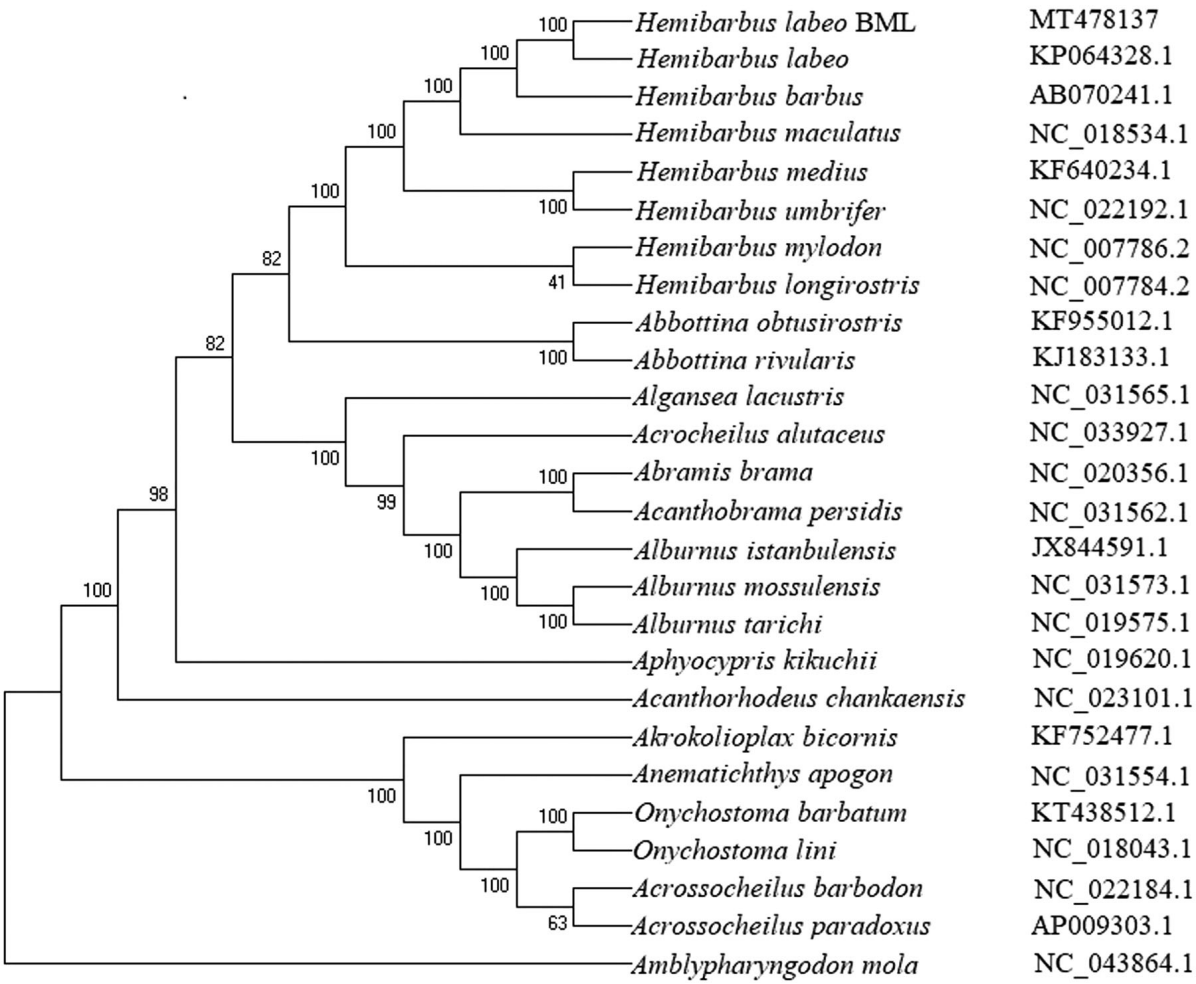
The phylogenetic analysis of *H. labeo* BML and other Cyprinidae fishes based on the mitogenome sequences.

## Data Availability

The data that support the findings of this study are openly available at NCBI (https://www.ncbi.nlm.nih.gov), GenBank accession no. MT478137. And the data that support the findings of this study are also available from the corresponding author, Dr. Yang, upon reasonable request.
